# Functional Evidence of Multidrug Resistance Transporters (MDR) in Rodent Olfactory Epithelium

**DOI:** 10.1371/journal.pone.0036167

**Published:** 2012-05-01

**Authors:** Adrien Molinas, Gilles Sicard, Ingrid Jakob

**Affiliations:** Equipe Neurophysiologie de la Peripherie des Systèmes Chimiosensoriels, Centre des Sciences du Goût et de l'Alimentation, CNRS UMR 6265, INRA, Université de Bourgogne, Dijon, France; Biological Research Centre of the Hungarian Academy of Sciences, Hungary

## Abstract

**Background:**

P-glycoprotein (Pgp) and multidrug resistance-associated protein (MRP1) are membrane transporter proteins which function as efflux pumps at cell membranes and are considered to exert a protective function against the entry of xenobiotics. While evidence for Pgp and MRP transporter activity is reported for olfactory tissue, their possible interaction and participation in the olfactory response has not been investigated.

**Principal Findings:**

Functional activity of putative MDR transporters was assessed by means of the fluorometric calcein acetoxymethyl ester (calcein-AM) accumulation assay on acute rat and mouse olfactory tissue slices. Calcein-AM uptake was measured as fluorescence intensity changes in the presence of Pgp or MRP specific inhibitors. Epifluorescence microscopy measured time course analysis in the olfactory epithelium revealed significant inhibitor-dependent calcein uptake in the presence of each of the selected inhibitors. Furthermore, intracellular calcein accumulation in olfactory receptor neurons was also significantly increased in the presence of either one of the Pgp or MRP inhibitors. The presence of Pgp or MRP1 encoding genes in the olfactory mucosa of rat and mouse was confirmed by RT-PCR with appropriate pairs of species-specific primers. Both transporters were expressed in both newborn and adult olfactory mucosa of both species. To assess a possible involvement of MDR transporters in the olfactory response, we examined the electrophysiological response to odorants in the presence of the selected MDR inhibitors by recording electroolfactograms (EOG). In both animal species, MRPs inhibitors induced a marked reduction of the EOG magnitude, while Pgp inhibitors had only a minor or no measurable effect.

**Conclusions:**

The findings suggest that both Pgp and MRP transporters are functional in the olfactory mucosa and in olfactory receptor neurons. Pgp and MRPs may be cellular constituents of olfactory receptor neurons and represent potential mechanisms for modulation of the olfactory response.

## Introduction

Among the numerous protein transporter systems present in many mucosal and epithelial tissue a few are toxically relevant efflux transporter proteins. They belong to the superfamily of binding cassette transporters (ABC transporters), transmembrane ATP-driven export pumps, and include amongst others two important members of two subfamilies, the P-glycoprotein (Pgp, gene symbol *ABCB1*) and the multidrug-associated resistance protein MRP1 (MRP1, gene symbol *ABCC1*). These proteins are especially well-known for their role in mediating the phenotype of multidrug resistance (MDR) seen in cancer cells where MDR is an acquired resistance against multiple chemotherapeutic drugs [1], [2]. Moreover these transporters are known to exert a protective role against toxicity of endo-and xenobiotics [3].

Beyond its inauspicious role in cancer treatment, Pgp has an important physiological function in normal tissue. In mammals, Pgp is constitutively expressed in cells specialized in selective or secretory transport, and generally in all tissues in which a highly selective compartmentalization is needed, such as kidney, pancreas, adrenal, glands, colon, jejunum, liver, brain [4]. Pgp has also been detected in tissues with barrier functions, such as in endothelial cells of the blood-brain barrier [5]. MRP1 ubiquitously distributed in tissue [6] is like Pgp an organic anion transporter protein, albeit with a different substrate profile. Pgp being a transporter with broad specificity [7] transports large hydrophobic positively charged molecules out of the cell, while preferred substrates of MRP1 are conjugated organic anionic compounds, some peptides and neutral compounds conjugated to glutathione, glucuronate or sulfate. Due to its ubiquitous presence in normal cellular physiology, various physiological functions including tissue defense against xenobiotic and endogenous toxic metabolites [8] are proposed.

Peripheral sensory systems like olfaction and taste localized at the interface between environment and body possess natural physical cellular structures with barrier characteristics [9] but are further endowed with a protective biochemical barrier. Detoxifying enzymes are particularly important in epithelia exposed to xenobiotic chemicals. The nasal tissue, especially, at the entry route to the CNS, has provided its different mucosal epithelia with several xenobiotic metabolizing enzymes [10]–[12] transforming xenobiotics into metabolites that are more readily absorbed. Multidrug transporters like Pgp and MRP1 are also expressed in nasal tissue, as well in respiratory and olfactory epithelia [10], [12]–[14]. The olfactory epithelium, segregated from the respiratory epithelium, lines the dorsal part of the nasal cavity harboring the olfactory receptor neurons (ORNs). Their specialized cilia are confronted with external cues which are beneficial or detrimental to the receiving organism. ORNs are first-order neurons, surrounded by supporting or sustentacular cells forming the apical layer of the olfactory mucosa. The underlying biochemical events of odorant detection and signal transduction are now well elucidated [15], [16].

In olfactory epithelium (OE) of bovine and mouse [17], [18]. Pgp has been localized to the apical cell layer, most likely to sustentacular cells. Also a very robust immunolocalization of MRP1 to sustentacular cells of the rat OE has recently been shown by Kudo et al. [19]. Further expression of MRP1, MRP3, MRP 4 and MRP 5 has been reported in the olfactory epithelium of rat [14], [19], [20]. The few functional MDR activity tests in OE of mammals have focused on Pgp transport activity across excised olfactory tissue or whole animals, and so far no functional test for MRP1 has been performed. Transporter activity of Pgp and MRP has been shown in OE of *Xenopus* tadpoles expressing the two transporter systems in sustentacular cells and olfactory neurons [21].

In the present study we assessed the function of the two transporters Pgp and MRP1 involved in multidrug resistance in the olfactory epithelium and compared two animal species. The calcein-AM assay is one of the best choices to probe MDR activities [22], [23] and analysis of fluorochrome efflux in combination with pharmacological tools is the major indicator of their expression and function. We confirmed MDR transporter expression in the OE by reverse transcription–PCR analysis and revealed constitutive expression of Pgp and MRP1 in newborn and adult animals.

To investigate whether MDR transporters may be directly or indirectly implicated in the olfactory response we conducted electrophysiological analysis of odorant-evoked responses by electroolfactogram recordings (EOGs) which measure mainly the odorant induced generator potentials thus reflecting initial stimulus-induced events from the periphery.

## Materials and Methods

### Animal treatments

All procedures involving animals were conducted in accordance with a protocol approved by the Local Ethics Committee for Research on Animals of the University of Bourgogne (France) under the approval number B1910. The care and husbandry of animals was in conformity with guidelines of the European Communities Council directive of Nov, 1986, (86/609/EEC). Adult Wistar rats and BALB/c mice of both sexes (2 to 7 months old) were purchased from Janvier, Le Genest-Saint-Isle, France, mouse and rat pups were provided by the local animal facility. Adult animals were deeply anesthetized by carbon dioxide inhalation, rat and mouse pups by hypothermia, and sacrified by decapitation.

### Calcein accumulation assay

A specific test for multidrug transporter-mediated activity is to measure intracellular calcein accumulation in the presence or absence of MDR inhibitors. Calcein acetoxymethlyl ester (calcein-AM) is a lipophilic, nonfluorescent dye that diffuses into cells where it is cleaved by cytosolic esterase to a green fluorescent dye. The amount of transporter activity is therefore inversely proportional to the accumulation of intracellular calcein fluorescence. Imaging experiments with calcein-AM were performed with a monochromator-based imaging system (Polychrome V, Till Photonics, Graefelfing, Germany) attached to an upright microscope (Olympus BX51WI) via a light guide and viewed with a x10 water immersion objective (UMPlanFl, NA 0.30). Fluorescent images were acquired with an IMAGO QE cooled charge-coupled device camera (PCO AG, Kelheim, Germany) controlled by the TILLvisION 4.0 software (TILL Photonics). The excitation wavelength for calcein was 480 nm and emitted light was collected by an appropriate dichroic mirror. Coronal slices of 100 and 150 µm thickness were prepared from olfactory epithelium of newborn mice and rats at postnatal day 0 to 2. Rapid hardening of the nasal bones and cartilage prevented exact acute slicing in older animals. Sections of the middle to dorsal portion of the nasal cavity where the olfactory bulbs are just apparent were cut on a vibrating slicer (Leica VT 1000 M/E, Nussloch, Germany) while submerged in cold standard Ringer solution. Slices were stored at 4°C in Ringer until transfer to an immersion chamber for optical recording at room temperature (RT).

A calcein accumulation experiment was performed in individual but serially adjacent slices from one animal, yielding normally 2–4 slices. As one slice was incubated with calcein–AM alone serving as control, thus allowing each experiment to have its own control, the remaining “sister” slices were loaded each with calcein-AM concomitantly one inhibitor. Calcein-AM concentration was in all instances 1 µM.

Typically one to three of the different inhibitors were studied in slices from one animal. A pre-incubation period of 8 min was necessary before fluorescence recording could be started. Thereafter images were recorded in a time series from 0 (t = 0) to 60 min (t = 60) at fixed 15-min intervals over 60 minutes of experiment. Calcein accumulation was measured as averaged pixel intensity inside of regions of interest (ROIs) for each time point in the 60 min sequence. For evaluation at the tissue level ROIs were delineated over an area extending over the entire height of the olfactory epithelium. Areas of different size and shapes or position gave essentially the same information and in each ROI no distinction was made between populated or non-populated pixel areas. Rates of change over time were calculated from slopes by linear regression. In addition, fluorescence intensities at the intracellular level of single cells were evaluated from fluorescence images (snapshot) at the 60 min end-point where small ROIs (8–12) were placed over the cell bodies of single cells. Ratios of inhibitor-to-control fluorescence intensities were than calculated from corresponding end-point images of the same series. Background fluorescence was subtracted in all recordings and determined using ROIs of identical shape and size as for evaluation, but placed in a cell-free area of the image.

### Electroolfactograms

We recorded electroolfactograms (EOGs) in the submerged field potential recording technique adapted from [24] with some modification. The severed head was cut in the mid-sagittal plane to expose the nasal septum in its totality, or after its careful excision the underlying endoturbinates. We recorded either from matched locations on the septum in rat and mouse, and additionally from a location in turbinate IIb of rat. Recordings were done by means of an Ag/AgCl wire in a disposable plastic gel loader tip filled with conductive gel (WPI, UK) and an Ag/AgCl disc positioned under the head serving as reference electrode. Both were connected to a custom-built differential amplifier. Signals were low pass filtered, sampled at 100 Hz, and digitized via a data-acquisition board (National Instruments, France) run by custom-made software on a personal computer. Data were later analyzed using Clampfit 8 (Axon Instruments, DIPSI, France) and other commercial software. The olfactory region was continuously superfused with a thin film of standard Ringer solution fed by gravity at a flow rate of 9 mL/min. Test stimuli were applied via a valve-operated loop (Rheodyne, Bioblock, France) with a volume of 120 µL allowing direct switching to the desired test solution.

### Solutions and chemical stimuli

The standard Ringer consisted of (in mM) 140 NaCl, 5 KCl, 2 CaCl_2_, 2 MgCl_2_, 10 HEPES adjusted to pH 7.2–7.3 and to 310 mosm with glucose. Ringer containing the appropriate concentration of transporter inhibitor are referred to as inhibitor-Ringer. MDR transporter inhibitors used were verapamil, cyclosporin A, probenecid, and MK571, prepared as 1 M stock solutions in Ringer except for probenecid which was dissolved in a diluted solution of NaOH buffered to pH 7.3 and prepared on the day of the experiment. Cyclosporin A was prepared as 25 mM stock solution. The following odorant and test stimuli were used: isoamyl acetate and 2,5-dimethyl pyrazine as single/pure odorants, and a mixture composed of isoamyl acetate, benzaldehyde, 2-heptanone, octanal, 1-octanol, acetophenone, octanoic acid, and 1,8-cineole. Odorants were prepared as 1 M stock solutions, 3-isobutyl-1-methyl-xanthine, (IBMX) and the mixture as 10^−1^ M stock solutions in dimethylsulfoxide (DMSO), elevated K^+^solution was the same as Ringer except that NaCl was replaced by 140 mM KCl. Final dilution in Ringer or inhibitor-Ringer to the desired concentration was freshly made before each experiment. Stimuli were diluted either in Ringer or inhibitor-Ringer. Test stimuli concentrations are reported as ‘loop’ concentration and not corrected for further dilution in the bath. Final DMSO concentration was maximally 0.01% or less with exception of the IBMX solution containing 0.1%, giving rise to a small EOG contributing to 3–5% of the maximal IBMX-induced EOG amplitude. Calcein-AM was prepared as 1M stock solution in DMSO and diluted to a final concentration of 1 µM in standard or inhibitor-Ringer at the start of the experiment. All experiments were performed at room temperature (20°–22°C).

### Olfactory modulation experiments

Modulation experiments involved the acquisition of three successive series of EOG recordings evoked by a standard stimulation protocol consisting of a set of stimuli sequentially presented to the olfactory mucosa. We acquired (1) an initial control series of EOGs in standard Ringer solution, (2) after having switched to inhibitor-Ringer for 10 min, a second series of EOGs in the presence of the desired inhibitor and stimuli prepared in inhibitor Ringer, (3) a third series of EOGs in standard Ringer was started after a 20 min period of washout with standard Ringer. This procedure was used for all inhibitor experiments, except that MK571-containing Ringer was superfused for only 3 min. The stimulus protocol consisted of sequentially applying KCl, IBMX, the odorant mixture, isoamyl acetate and 2, 5-dimethyl pyrazine. With the exception of KCl, the stimuli were presented at a concentration of 10^−5^ and 10^−4^ M. Inter-stimulus interval was at least 2 minutes. Peak amplitude of the EOG response was measured as the maximum negative voltage deflection from baseline. For comparison of response kinetics EOG waveforms were normalized to their peak amplitudes. EOG rise times were defined as the time of the start to the peak of the response. For comparison the rates of rise were fitted to a sigmoidal function and the half-maximal activation (V_50_) of treated and control EOGs compared by a paired *t*-test. For comparison of decay time waveforms were aligned on the peak of the amplitude and inspected for disparities.

### RT-PCR on olfactory epithelium

Tissue samples of liver and main olfactory epithelium of adult (n = 2) and neonate (n = 2) animals of each species were rapidly removed into PCR tubes, snap frozen in liquid nitrogen and stored at−80°C until RNA extraction. To avoid contamination with respiratory cells only the caudal/ posterior one third of the olfactory mucosa of the septum was peeled off from the cartilage thereby avoiding also the area near the choanae (herein after referred to as simply olfactory mucosa). Total RNA was extracted from frozen tissue using RNeasy™ Plus mini Kit (Qiagen, France) according to the manufacturer's instructions. The quantity and purity of RNA was determined with a spectrophotometer (Nanodrop 2000 Thermo Scientific). 1.3 µg RNA was reverse transcribed using Superscript III First strand Synthesis System for RT-PCR (Invitrogen) and oligo dT primers in a UNO II Thermocycler (Biometra). PCR reaction was performed using standard protocols with TaqPCR Master Mix (Qiagen, France). PCR amplification was started with an initial denaturation in one cycle at 98°C for 60 s, followed by denaturation step of 98°C for 30 s, annealing at 60°C for 40 s, and extension at 72°C for 60 s, for a total of 35 cycles, followed by a final extension step at 72°C for 5 min. Negative controls for contamination by extraneous DNA (omission of reverse transcriptase) and RNA were run with each set of reactions. Positive controls were done by amplification of a cDNA fragment of cyclophilin A, a housekeeping gene. Amplified cDNAs were separated by electrophoresis in a 1.3% ethidium bromide stained agarose gel and visualized under UV light on a Molecular Imager Gel Doc XR System (Bio-Rad). The specificity of the PCR product was confirmed by sequencing.

Specific primers were designed by Blast searching using publicly available sequence information of the GenBank of the National Center for Biotechnology Information (http://www.ncbi.nlm.nih.gov/BLAST/) and purchased from Operon (Eurofins MWG Operon, Germany).

### Data analysis

All figures giving qualitative information represent typical results of at least three different experiments (stated when less). Statistical analyses were performed using a two-tailed paired ratio *t-*test [25] or one-sample *t*-test where appropriate. In both cases differences were considered significant at p<0.05. Linear regression analysis was carried out to analyze calcein uptake rates and linear relationships with R^2^ values greater than 0.95 were used. To detect far outliers Grubbs test was applied. All data analyses were carried out with GraphPad 4.0. Data are expressed as mean ± standard deviation (SD).

### Chemicals

Calcein-AM was purchased from Invitrogene (France), IBMX from Calbiochem (France), Verapamil and MK571 from Biomol (France), all other chemicals were obtained from Sigma Aldrich (St. Quentin, France).

## Results

### Functional testing of multidrug resistance transporter activity

A specific test for multidrug-transporter mediated activity (MDR) is to measure intracellular calcein accumulation in the absence and presence of MDR inhibitors. The assay is based on the use of lipophilic acetoxymethyl ester of calcein (calcein-AM), which crosses membranes. Once internalized and enzymatically converted to calcein, the dye becomes highly fluorescent. Both calcein-AM and calcein have been shown to be substrates for MRP, but only calcein-AM is a substrate for Pgp [23]. To examine the functional activity of MDR transporters we incubated olfactory tissue slices of newborn mice and rats with calcein-AM in the presence or absence of appropriate MDR inhibitors. Fluorescence data were obtained from 28 slices from mice (n = 16) and 30 slices from rats (n = 19).

**Figure 1:**
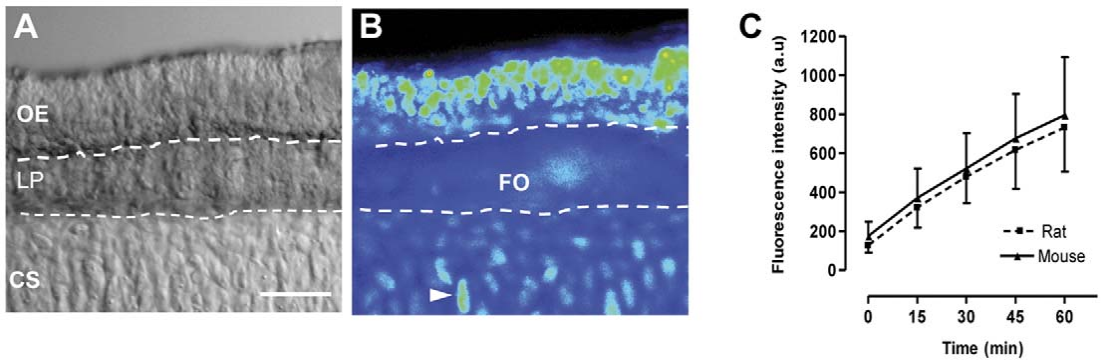
Calcein fluorescence pattern and accumulation in olfactory tissue slices of mouse and rat. A: Transmission light micrograph of a part of a typical coronal section through the main olfactory epithelium of mouse featuring three main layers: the olfactory epithelium (OE), the lamina propria (LP) and the nasal cartilage of the septum (CS). **B:** Pseudocolored fluorescence image of the same section after 30 minutes of incubation with 1 µM calcein-AM. Note that main staining occurred in the OE, some staining in the LP mainly in the fila olfactoria (FO), and cartilage cells, the chondrocytes (arrowhead). **C:** Time-dependent changes of calcein accumulation in the OE of rats (n = 19) and mice (n = 16) as determined from average fluorescence intensities of regions of interests (ROIs) at indicated time points. Fluorescence intensity in arbitrary units (a.u), scale bar in A = 50 µm.

Calcein-AM application led to an immediate but faint fluorescence rise in olfactory tissue slices, and was barely detectable during the first 8 minutes, not revealing morphological structures of the tissue. This made it difficult to report the increase in fluorescence during this time of incubation considered as pre-incubation. Thereafter, the time series experiment was started at time 0 and images sequentially acquired at fixed intervals of 15 min for a period of 60 min. Figure 1 B illustrates a representative fluorescence distribution pattern in a part of a coronal section through the mid-septal portion of the OE after incubation in 1 µM calcein-AM for a period of 30 min. Calcein fluorescence distribution was non-homogenous within the slice and developed primarly within the olfactory epithelium layer as indicated in the corresponding transmission micrograph (Figure 1A) showing the general morphological aspects of the same section with its three distinct main layers. The outer and upper layer represents the olfactory epithelium (OE), the subjacent layer, the lamina propria (LP), consists of connective tissue with nasal glands, blood vessels, and olfactory nerve/axon bundles (fila olfactoria, FO) which also accumulate calcein. These two layers overlay the nasal cartilaginous bone (CS) with its chondrocytes where a faint and patchy staining of calcein fluorescence was also observed. Generally similar patterns of fluorescence distribution for calcein appeared in mouse and rat slices. Calcein uptake was estimated from intensity changes in calcein fluorescence and evaluated in regions of interest (ROIs) from all five time points. Figure 1C shows the fluorometric time course of calcein accumulation over the 60 min experimental period into the OE of mice and rats in absence of MDR inhibitors. Fluorescence rose steadily and increased quasi linearly without saturation over the time of experiment. Average calcein fluorescence levels in the OE were similar in magnitude in both species but there was considerable variation in fluorescence uptake among individual specimens. Uptake rates calculated from slopes by linear regression ranged from 3.04–15.61 in rats and from 3.86–16.17 in mice (arbitrary units/time, a.u./60 min).

**Figure 2:**
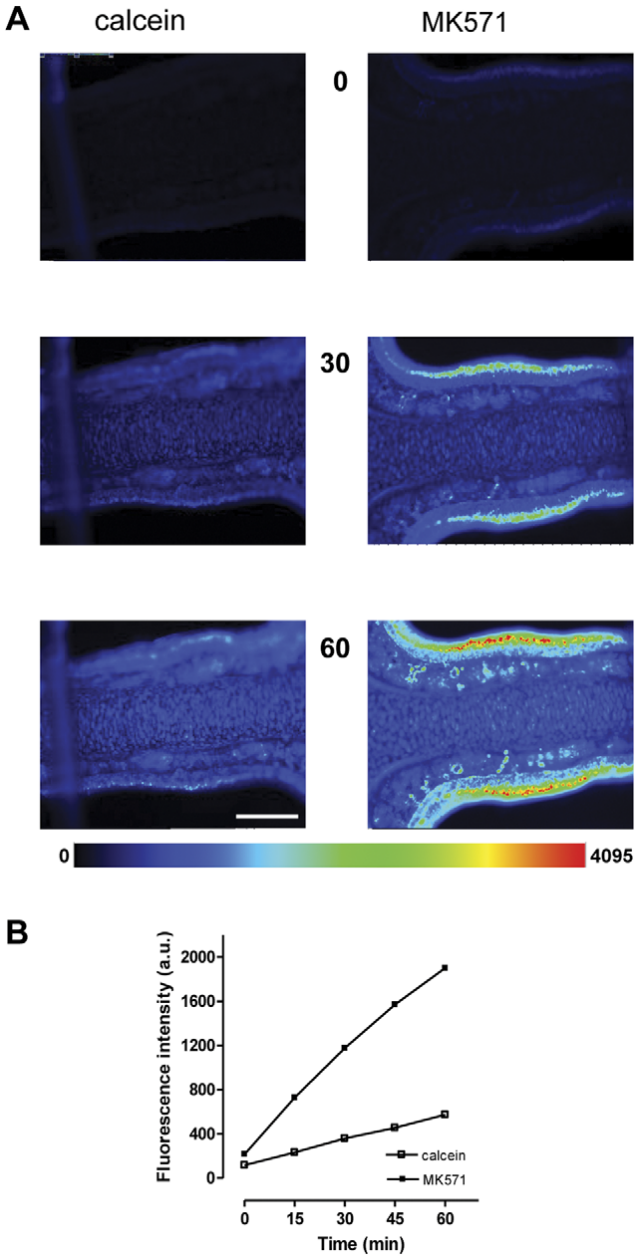
Calcein fluorescence in olfactory slices is elevated in the presence of an MDR inhibitor. A: Time series images in pseudocolor of two individual mouse olfactory slices incubated with 1 µM calcein–AM (left panel), or concomitantly with 25 µM MK571 (right panel). The numbered images correspond to individual frames at indicated time points. Some pixel saturation occurred at the 60 min time point in the center portion of the OE in MK-treated slice. The two slices are from the same specimen, recorded with identical exposure times. **B:** Time-dependent accumulation of fluorescence intensity (arbitrary units, a.u) for calcein in the absence or presence of 25 µM MK571 as evaluated from regions of interest (ROIs), saturated pixels were not considered for evaluation. Data derived from above experiment. Values of the pseudocolor code bar scales from 0 (low, blue) to 4095 (high, red), scale bar in A = 200 µm.

Next we evaluated the effect of various inhibitors of MDR transporters on calcein accumulation. The following four agents well recognized for their MDR reversing or inhibiting capacities were tested: verapamil and cyclosporin A are inhibitors of the Pgp transporter, while probenecid and MK571 are inhibitors of MRP-transporters. Direct observation by fluorescence microscopy revealed that inhibitor-incubated tissue slices always exhibited stronger fluorescence emission than when incubated with calcein-AM alone. This is illustrated in the example in Figure 2A for images of the 0, 30 and 60 min time points from mouse olfactory slices incubated with calcein-AM or concomitantly with MK571. When compared to the control with calcein, fluorescent visualization of the MK-treated slice demonstrated a progressively increasing change in brightness already at the starting time point 0 (after 8 min of pre-incubation). Brightness was pronounced at the 30 min time point, and at the 60 min time point fluorescence intensities in the MK–treated mouse slice covered the full range of the 4065 intensity levels approaching partially sub-saturation to slight saturation levels in a small band in the center of the OE. Saturation occurred under the given recording conditions in all mouse slices (4/4), but not in rat slices (0/4).

**Figure 3:**
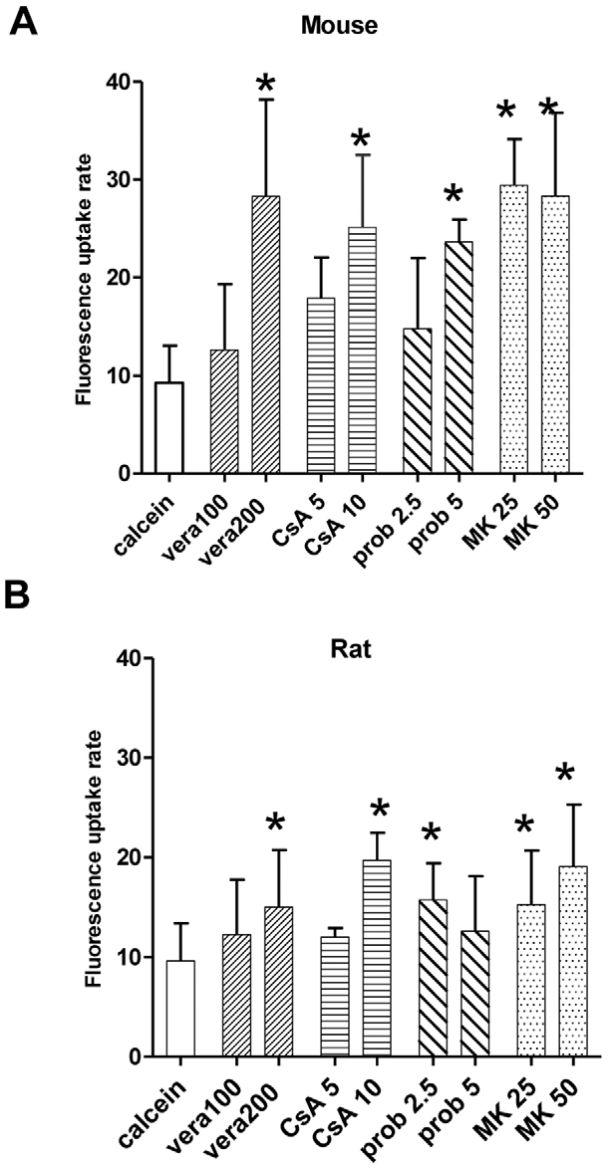
Effect of various MDR inhibitors on calcein accumulation in olfactory slices of rat and mouse. Olfactory tissue slices were incubated with 1 µM calcein-AM in the absence or presence of the specific MDR inhibitors. Bars represent average accumulation rates as determined from slopes over all time points of the experiment, in (**A**) from mice, and in (**B**) from rats. The following inhibitors were tested: vera  =  verapamil at 100 and 200 µM, CsA  =  cyclosporin A at 5 and 10 µM, prob  =  probenecid at 2.5 and 5 mM, and MK  =  MK571 at 25 and 50 µM. Values from at least three independent assays for each inhibitor and concentration. * p<0.05.

A more quantitative evaluation of calcein fluorescence changes was carried out from regions of interest (ROIs) over the whole height of the OE. The time-dependent fluorescence changes (of non-saturation levels) of the above experiment showed an almost linear increase over the 60 min period (Figure 2B) with a mean fluorescence intensity of 575 a.u. for the calcein control and 2000 a.u. for the MK-treated slice at the 60 min time point, a value that is 3.3-fold of that of the control value. The uptake rate, as calculated from slopes, was 28.07 for MK and 7.5 for calcein which represents approximately a4-fold increase. All of the four inhibitors tested increased calcein fluorescence in mice OE, but with different efficiencies. Figure 3 compares and summarizes the effect of inhibitors on the rates of calcein accumulation in mouse and rat slices. The increase was concentration dependant and the effect was statistically significant at the highest concentration tested, for verapamil at 200 µM, cyclosporin 10 µM, and probenecid at 5 mM. Figure 3A also shows that increasing the concentration of MK from 25 to 50 µM in mouse slices did not further increase fluorescence intensities. In rat slices, all inhibitors tested had a clearly visible fluorescence effect, but calcein accumulation during inhibitor application was in general less than in mouse slices (Figure 3B). MK was significantly effective at both concentrations of 25 and 50 µM and cyclosporin at 10 µM. Probenecid was significantly effective only at 2.5 mM. In both species transporter inhibition by MK induced the highest uptake rate, followed by cyclosporin. The experiments described above demonstrated a significant effect on calcein accumulation by Pgp and MRP-dependent inhibitors and the results suggest the presence of at least two functional MDR transporters in the OE.

**Figure 4:**
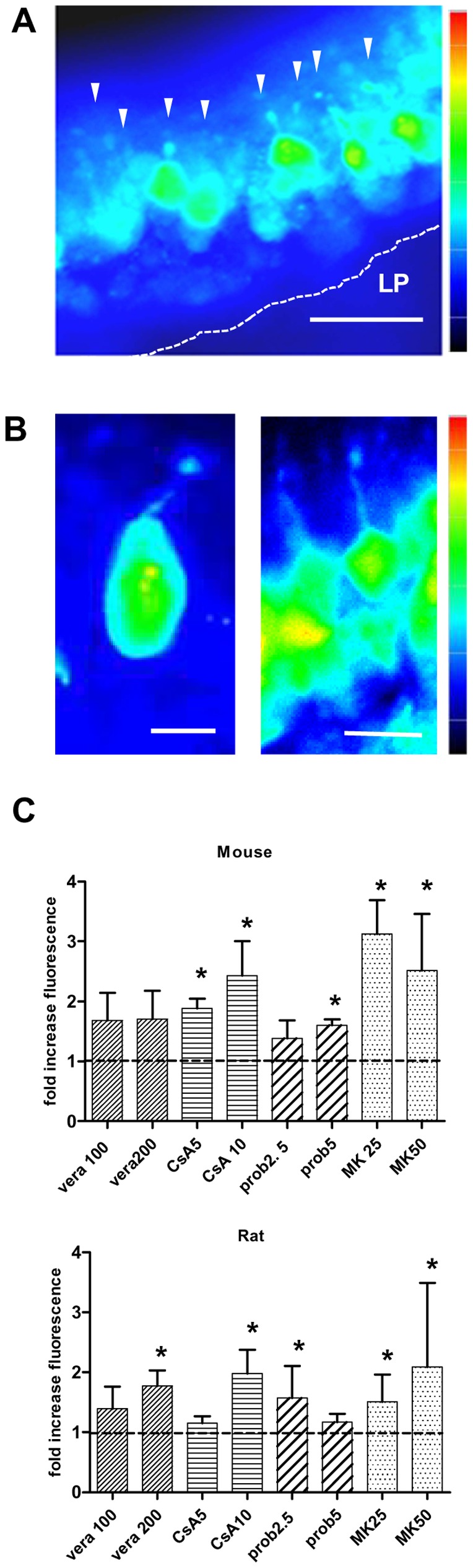
MDR inhibitors enhanced calcein accumulation in olfactory receptor neurons (ORNs) of mouse and rat. A: Pseudocolored fluorescence micrograph of a part of an olfactory slice from rat in the presence of MK571 (50 µM) at the 60 min time point. Arrowheads point to ORNs appearing as pear-shaped cells with visible knob-bearing dendritic structures. Fluorescence image adjusted for contrast, pseudocolor bar scales from low (blue) to high (red) intensities. LP  =  lamina propria, scale bar in A = 20 μm. **B:** Fluorescence scaling is different for the two images, the value of the pseudocolor bar scales 0 to 1500 for the left panel and micrograph of a single mouse ORN in the presence of calcein-AM (left panel) and a row of ORNs in the presence of cyclosporin A (right panel). Both images are taken at the 60 min time point. Note, that the 0 to 4095 for the right panel. Scale bar in the left panel = 5 µm, and 10 µm in the right panel. **C:** Intracellular inhibitor-dependent increase in fluorescence intensities was evaluated from regions of interest (ROIs) placed over the cell bodies of ORNs at the 60 min time point. Bars represent the averaged inhibitor-to-control ratios of calcein fluorescence intensities in mouse (upper panel) and rat (lower panel) for four tested inhibitors: vera  =  verapamil at 100 and 200 µM, CsA  =  cyclosporin A at 5 and 10 µM, prob  =  probenecid at 2.5 and 5 mM, and MK  =  MK571 at 25 and 50 µM. * p<0.05.

Because the whole surface of the OE was incubated and exposed to calcein-AM, the above method measured the tissue response from a heterogeneous cell population in the OE and did not distinguish individual cell types. The OE consists of at least three different cell types organized in horizontal layers, but due to a general lack of contrast of the conventional fluorescence image it was not possible to reveal the exact tissue and layer boundaries. The small narrow band in the center of the OE, however, could be identified as the layer populated by the cell bodies of olfactory receptor neurons (ORNs). Within this layer details on cell morphology and dendritic knob-bearing structures became best detectable by the end of the experiment at the 60 min time point as shown in Figure 4A. This made it possible to evaluate inhibitor-dependent calcein accumulation in single ORNs (Figure 4B) from end-point images of before described recordings. We calculated ratios of intracellular fluorescence accumulation in single ORNs in the absence and presence of the four tested inhibitors. MK had the largest inhibitory effect and significantly increased calcein in mice ORNs about 3-to 4fold and 2-to 3-fold in rats (Figure 4C). In mice ORNs, probenecid at 5 mM, and cyclosporin at both tested concentrations was also effective. In rats, probenecid was only significantly effective at a concentration of 2.5 mM, cyclosporin and verapamil at the highest concentration tested. In some instances we were also able to evaluate the fluorescence changes at the 60 min end-point for a small number of sustentacular cells. Cells were selected on morphological criteria and evaluated for fluorescence changes in presence of 10 µM cylosporin or 50 µM MK571. As for ORNs, MK appears to have the largest inhibitory effect increasing fluorescence about 2.5-fold in mouse and 3.4-fold in rat sustentacular cells while fluorescence increase with cyclosporin was 2-fold for mouse and rat (not shown).

### Presence of *mdr1a*, *mdr1b* and *mrp1* mRNA in olfactory tissue

**Figure 5:**
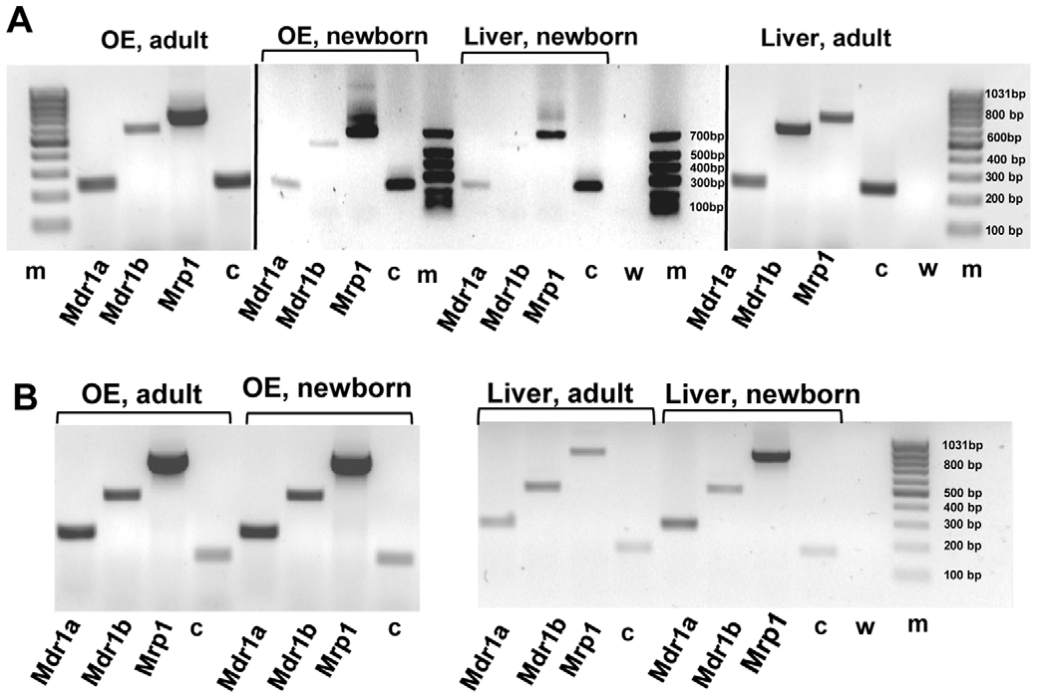
Distribution of mdr and mrp mRNAs in rat and mouse olfactory and liver tissue. The presence of *mdr1a*, *mdr1b* and *mrp1* mRNA analyzed by means of RT-PCR from samples of olfactory mucosa and liver tissue. Electrophoresis of PCR products **A:** from newborn and adult rat, **B:** from newborn and adult mouse. The cDNAs were amplified with rat- and mouse-specific primers for the Mdr1a, Mdr1b and Mrp1 genes. All tissues were positive for the tested cDNAs (note that staining in A, lane 12 for *mdr1b* is weak, but visible on the gel). c  =  cyclophilin A used as positive control, w  =  negative control containing water in place of cDNA, m = DNA size markers. All gels were run together except for the newborn rat, which was run on a separate gel. Results from a single experiment.

To further corroborate the results of the functional activity of multidrug transporters, RT-PCR was undertaken to confirm whether the encoding gene for drug-efflux conferring multidrug resistance transporters P-glycoprotein (Pgp) and multidrug resistance-associated protein (MRP) were expressed in olfactory mucosa. We tested for mRNAs of the two P-glycoprotein isoforms, Mdr1a and Mdr1b, and Mrp1 in olfactory mucosa and liver tissue in newborn and adult rats and mice. To ensure olfactory tissue purity we took great care to avoid respiratory epithelium contamination in our samples. Specific primer pairs for the detection of the mouse and rat *mdr1a*, *mdr1b* and *mrp1* cDNAs (Table 1) were used. These primers were designed to amplify cDNA from mRNA and not genomic DNA sequences. Agarose gel separation of the amplification products showed cDNA fragments of the expected size (Table 1) for all three transcripts. All samples of rat and mouse tissue tested expressed *mdr1a*, *mdr1b* and *mrp1* (Figure 5 A, B). Cyclophilin A was used as a positive RT-PCR control (c in Figure 5 A, B). As expected, no bands were detected in the negative control, which consisted of replacing cDNA templates with water (in Figure 5 A, B). PCR products were sequenced to confirm identity of the amplicons. The analysis revealed the presence of mRNA for both isoforms of P-glycoprotein, Mdr1a and Mdr1 b, and Mrp1 which indicates that the genes are very likely expressed in olfactory mucosa and liver of newborn and adult animals. This expression pattern persisted throughout the developmental period into adulthood.

**Table 1:** Primer pairs used for the amplification of cDNAs for rat and mouse Mdrs and Mrps.

	Rat			
Gene		Primer (5′-3′)	Genbank Accession No	Amplicon (bp)
MRP1 (Abcc1)	forward	ATGTGACTCTCAAGGGCTCCGTGG	NM_022281	691
	reverse	GTGCTGCTGTGCTGCTGGTTAGTA		
MDR1a (Abcb1a)	forward	GAGTGAAAAGGTCGTCCAGGAAGCG	NM_133401	234
	reverse	TCTCGCATGGTCACAGTTCATGAGC		
MDR1b (Abcb1b)	forward	CCCAAAGTGACACTGGTGCCTCTG	NM_012623	564
	reverse	GCCTGGAGCCCATAGCCCCTTTA		
Cyclophilin A (CypA)	forward	TGGCACTGGTGGCAAGTCCA	NM_017101	229
	reverse	TGGACCCAAAACGCTCCATGGC		

	Mouse			
Gene		Primer (5′-3′)	Genbank Accession No	Amplicon (bp)
MRP1 (Abcc1)	forward	AGCATTGTGCAGGCCAGCGT	NM_008576.3	822
	reverse	CTCCGAGGCCAGGTCCTGCT		
MDR1a (Abcb1a)	forward	CCTGAAACCCAGCGGCAGAACA	NM_011076.3	265
	reverse	GCCTAGACCCTGTAGCCCCTTTCAC		
MDR1b (Abcb1b)	forward	GTGTCCACAGAAAGCAAGACCAAGA	NM_011075.3	482
	reverse	GGCGCCCATCGCCCCTTTAA		
Cyclophilin A (CypA)	forward	CATCCTAAAGCATACAGGT	NM_008907	165
	reverse	TCCATGGCTTCCACAATGTT		

### Modulation of the olfactory response

To test the hypothesis that MDR activities may be involved in the peripheral processing of odorants we directly assessed the function of the olfactory neurons by recording stimuli evoked electroolfactograms (EOGs) in the absence and the presence of MDR inhibitors. We sampled EOG responses from two olfactory mucosal surfaces, the septum (n = 18) and the turbinate (n = 18) in rat, and from a matching recording site in the mouse septum (n = 20). A series of chemical and odorant stimuli were presented, consisting of KCl, IBMX, an odorant mixture, and two different pure odorants, isoamyl acetate and 2,5-dimethyl pyrazine. IBMX is a phosphodiesterase inhibitor thus increasing cAMP which leads in turn to cyclic nucleotide channel (CNG) activation. KCl activates voltage-dependent channels i.e. Na^+^ and Ca^2+^ and is a measure of the general excitability of the preparation.

**Figure 6:**
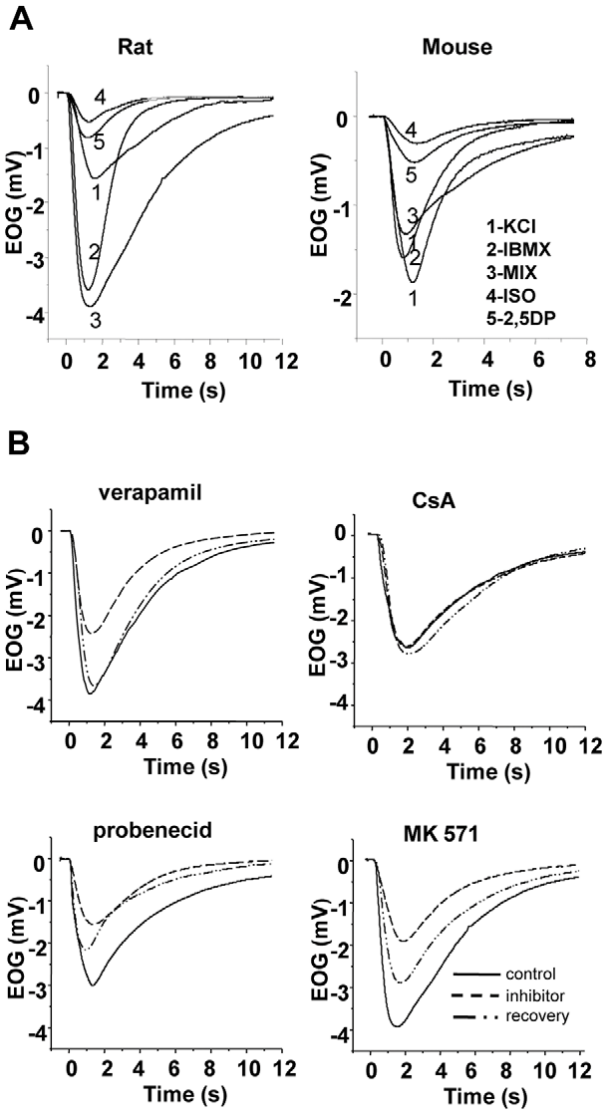
Modulatory effect of MDR inhibitors on the olfactory response to odorants in rat and mouse. **A:** Representative superimposed electroolfactogram traces recorded in the septal olfactory mucosa of a rat (left) and a mouse (right) under control conditions. Responses are recorded to a sequence of stimuli (inset, left) as KCl, IBMX, a mixture of odorants, isoamyl acetate and 2,5-dimethyl pyrazine, at concentrations of 10^−4^ M. **B:** Representative selection of EOG recordings from rats in response to the mixture at a concentration of 10^−4^ M before (control, solid line), during (inhibitor, dashed line), and after (recovery, dot-dash line) application of four MDR inhibitors: verapamil at 200 µM, cyclosporin A (CsA) at 5 µM, probenecid at 2.5 mM and MK571 at 25 µM. Recordings are from four different specimen.

Representative control EOG traces evoked by the series of stimuli at a concentration of 10^−4^ M recorded in the septum of rat and mouse, respectively, are represented in Figure 6A. Qualitative similar recordings were also obtained from the recording site in the turbinate of the rat. As expected EOG waveforms varied with stimuli, as well as with animals and the recording location. All responses were characterized by monophasic negative waveforms with a rapid rise to peak and a slower return to baseline or near to the baseline. With the exception of KCl, all stimuli were tested at two increasing concentrations, at 10^−5^ and 10^−4^ M. Response magnitude was concentration-dependent (not shown) and the relation between the peak amplitude of the two concentrations varied between 1.8 and 3.2-fold depending on the substance. Odorant responses were largest in the septum of the rat. Mean peak amplitude evoked by the mixture at a concentration of 10^−4^ M was 3.92±0.85 mV ranging from 2.55–5.46 mV, while isoamyl acetate at the same concentration produced a mean amplitude of 1.28±0.56 mV ranging from 0.61 to 2.73 mV. Compared to rats, all stimuli induced-response magnitudes in mice were generally, approximately between 64–72 % smaller (Figure 6A), i.e. the mixture-evoked mean peak amplitude for the concentration of 10^−4^ M was 1.18±0.36 mV ranging from 0.52–1.77 mV. For the modulation experiments the four different previously tested inhibitors were applied at the following concentrations, verapamil at 200 μM, cyclosporin at 5 μM, probenecid at 2.5 mM, and MK at 25 μM, additionally cyclosporin was tested at a concentration of 10 μM, and probenecid at 5 mM in mice. Figure 6B illustrates the effects of the four different inhibitors on the mixture-evoked response in the rat septum. In these recordings verapamil, probenecid and MK reduced the peak amplitude to between 40–52%, while cyclosporin had no measurable effect. Similar reductions of amplitude but with varying degree of inhibitions were observed with all other stimuli, also in rat turbinates and mouse septum. The effects were reversible after initial reduction as peak amplitudes were restored to 50–100 % after washout of the inhibitor.

**Figure 7:**
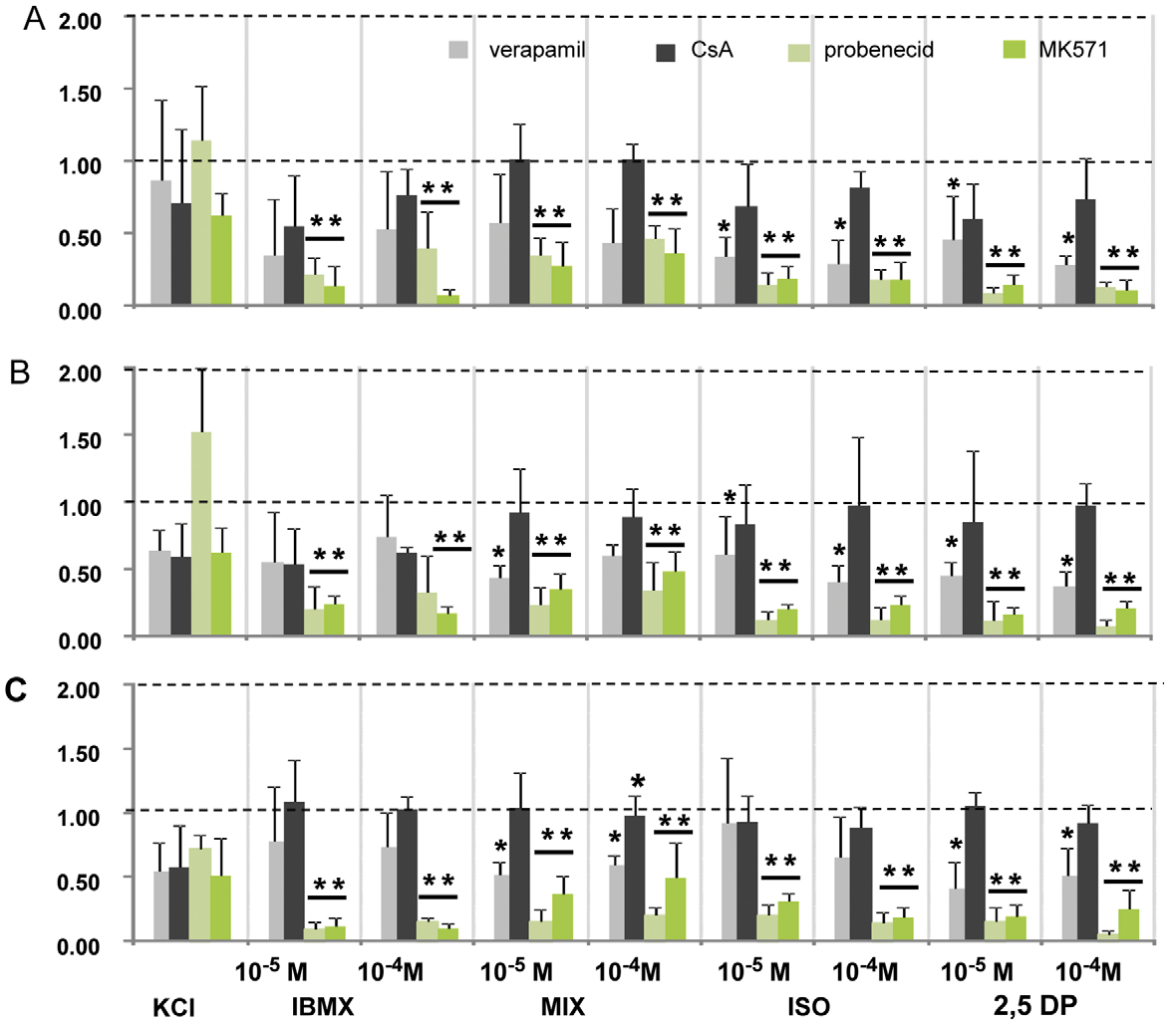
Summary of the modulatory effects of four MDR inhibitors on olfactory response amplitudes. **A:** in the rat olfactory septum, **B:** in the rat turbinate, and **C:** in the mouse olfactory septum. Bars represent normalized average peak amplitudes. Responses were normalized with respect to control before inhibitor application which was set to unity. The following stimuli were sequentially applied in control condition and in presence of the specified inhibitor: KCl, IBMX, a mixture of odorants (MIX), isoamyl acetate (ISO), and 2,5-dimethyl pyrazine (2,5DP), at the indicated concentrations. The inhibitors tested were verapamil at 200 µM (gray), cyclosporin A at 5 µM (black), probenecid at 2.5 mM, (light green) and MK571 at 25 µM (green). Note that for the mouse (in C) the results for 5 mM probenecid are shown. **p<0.001, * p<0.05.

Because the absolute magnitude of a response to a stimulus varied between different preparations and specimens, the amplitude of each stimulus was normalized to its control response before inhibitor application. The averaged normalized amplitudes in the presence of the four tested MDR inhibitors were compared in Figure 7. In general KCl responses were less sensitive to all tested inhibitors independent of the recording site. Verapamil (200 μM) had a moderate decreasing effect on all responses in rat and mice, but this was not consistently significant, only for isomayl acetate and 2,5-dimethyl pyrazine in the rat septum and turbinate. In the mouse septum only the response to the mixture and 2,5-dimethyl pyrazine was significantly decreased. The presence of 5 μM cyclosporin decreased slightly the response amplitudes in all three recording sites, but the effect was not significant. Increasing its concentration to 10 μM (n = 2) remained also without significant effect. Of the four compounds tested, the MRP specific inhibitors MK571 and probenecid introduced the strongest reduction. The responses to all stimuli in all three recording sites were dramatically and significantly reduced by MK (52–94 %) at a concentration of 25 μM. Probenecid at 2.5 mM caused similar significant reductions in all responses in rat but was less effective in mouse EOGs. While a strong effect was already observed at 2.5 mM probenecid in mouse EOG responses, 5 mM were required for a reduction of 85 to 95% for all stimuli-induced responses.

**Figure 8:**
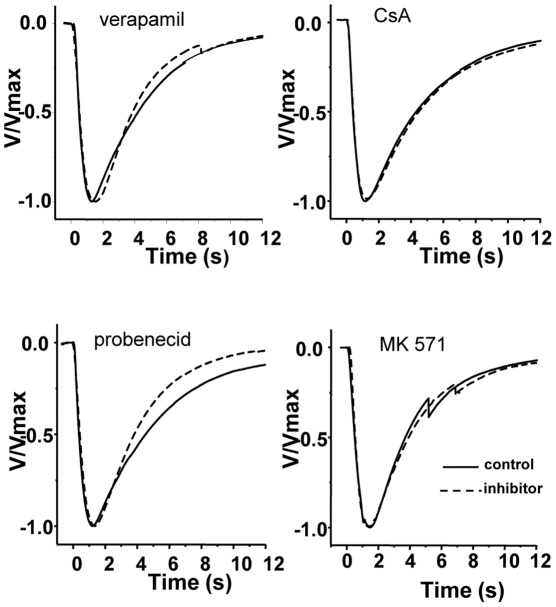
Comparison of the kinetics of olfactory responses during inhibitor application. Representative electroolfactogram (EOG) traces from rat in response to the odorant mixture of 10^−4^ M before and during MDR inhibitor application. EOG traces were normalized to their maximum amplitude and averaged for the comparison of their time course. Inhibitors applied were verapamil at 200 µM, CsA  =  cyclosporin A at 5 µM, probenecid at 2.5 mM, and MK571 at 25 µM. Each averaged waveform represents the independent assessment from four different animals.

To uncover disparities in the shape of EOG waveforms, we normalized all recordings to their peak amplitudes, superimposed and aligned them on their onset. A representative sample is shown in Figure 8 for the effect of the four tested MDR-inhibitors on the averaged mixture-induced responses in the rat septum. Inspection of the superposition of all 108 pairs (9 pairs/ inhibitor/ recording site, n = 4) did not reveal any distortion of the general aspect of the waveform, but subtle differences in activation kinetic. In approximately 65% (61/93) of the inhibitor-control pairs the time course of the rising phase was nearly identical and did overlap to almost 100 %, the remaining 35 % showed a shift to slower rise times. We further analyzed the rates of rise by fitting the normalized responses to a sigmoidal curve (not shown) and measured the half- maximal activation (V_50_). Although the shift to slower rise times was present in all of these recordings, it was statistically not significant. To compare decay kinetics waveforms of inhibitor-control pairs were aligned on their peak amplitude and the overlays inspected for differences. Decay kinetics were not systemically affected. In 40/90 waveform pairs no change occurred, while 35/90 had slightly slower and 15/90 faster decay kinetics, although there was a tendency for verapamil and probenecid to show faster decay kinetics on the rat septum. This was independent of stimuli or inhibitor used. Though the peak amplitudes of the voltage responses were different, the overall shape of the waveform was only modestly changed or stayed comparable to that of the control.

## Discussion

The results of our study demonstrate MDR transporter activities in the olfactory epithelium and provide comparative data on the function of Ppg and MRP transporters for two animal species, the mouse and the rat. Using a fluorometric assay in combination with pharmacological inhibitors we show that both transporters are functional in the olfactory epithelium, and moreover in olfactory neurons. Electrophysiological recordings from the OE indicated that the response to odorants was reduced when MDR activity was inhibited thus revealing their activity.

Functional assays to detect MDR transporter activity involve the use of fluorescent transporter substrates such as calcein-AM [26], [27]. While typically the fluorescent assay is used for detection of MDR transporters in MDR overexpressing cells, the assay has already been proven to be suitable in physiological acute slice preparations of rodent taste cells and *Xenopus* olfactory epithelium [21], [28]. The assay is based on the principle that active MDR transporters will restrict calcein accumulation in the tissue where they reside resulting in low fluorescence. This activity can be reversed in the presence of specific inhibitors thus enhancing fluorescence intensity. Our functional studies show that calcein accumulation in rodent OE is significantly enhanced in the presence of potent Pgp and MRP inhibitors. To increase the probability of detecting transporter activity, two pairs of structurally diverse inhibitors were selected appropriate to the transporter type. The calcein fluorescence readily developing in the OE in the absence of any of the selected inhibitors argued, in a first instance, against a functional expression of MDR transporters in the OE. But all four selected inhibitors, however, significantly increased calcein fluorescence in the OE in a time- and concentration-dependant manner, thereby revealing similar inhibitor profiles for both animal species with small differences in sensitivity among them with probenecid being already saturated at the lower concentration. To make appropriate interpretation of inhibitor interaction data the specificity of the inhibitors has to be considered. The Pgp inhibitors cyclosporin and verapamil have been proven to be reversing agents in ‘physiological’ sensory cells [21], [28] and in cells transiently transfected with Pgp [7]. In a large *in vitro* study on recommendation for inhibitor/probe use, cyclosporin and verapamil have been classified as good Pgp inhibitors [29]. Considering that verapamil is a known calcium channel blocker, it may or may not exert this function in the calcein-AM assay as calcein fluorescence is insensitive to changes in cytosolic calcium [30]. MK571 has been previously defined as a specific blocker of MRP1 [31] but also inhibits other MRP species. Probenecid is an inhibitor of organic anion transporters acting on MRP isoforms other than MRP1, but not on Pgp [32]. Other members of the MRP family as MRP4 are expressed [14] and MRP3 and MRP5 has been recently histochemically localized in rat olfactory receptor neurons [33]. MRP5 activity cannot be assessed with the assay used here as calcein is not a substrate [34], but we cannot exclude that MRP3 or MRP4 activity may contribute to calcein accumulation in our experiment. Assuming a high specificity of the selected inhibitors the increased calcein accumulation/retention is brought about by the activity of Pgp and MRP1 and/or MRP-like transporters. Both transporters, functional in the OE of newborn rodents, behaved in a similar manner in each species.

The most striking result of the present study was the modulation of MDR activity in single ORNs. Microscopical examination at the 60 min time point allowed to unambiguously identifying ORNs by their distinctive and unmistakable morphology in contrast to the more undescriptive sustentacular cells. Calcein uptake curves for all inhibitors tested were nearly linear over 60 min of experiments thus allowing for evaluation of calcein accumulation at the 60 min end-point. Cellular accumulation of calcein by Pgp or MRP inhibition was associated with an inhibitor-induced increase in fluorescence within single ORNs and comparison between single ORN and tissue fluorescence intensities revealed qualitative similarities in inhibitor profile. These findings suggest a functional activity of the two transporter systems in single rodent ORNs. Very similar results have been obtained for larval *Xenopus* ORNs [21]. Further support to our findings comes from immunolocalization and antisense signaling of MRP1 [14], [19] both reporting the presence of MRP1 mRNA in ORNs of adult rat. More intriguing is the finding that MRP1 is most likely expressed in olfactory cilia of rats [35], the site where initial olfactory transduction takes place. In contrast to the presence of MRP1, no Pgp immunological expression is reported for rat olfactory mucosa. In a recent immunohistological study Thiebaud et al. [33] localized Ppg expression only to a vessel in the submucosa. The discrepancy may be explained by the method as the calcein–AM staining method is more sensitive than immunostaining. Then again, evidence based on an immunohistochemistry with the antibody C219, as used by [33] is very often compromised in paraffin and/or formalin treated tissue [36], [37]. For immunolocalization of Pgp often various fixation conditions and different antibodies are required as shown for rat taste buds or hippocampus neurons of rat [28], [38].

Reports on localization or activity of MDR transporters in normal physiological neurons are yet scarce. Normally present in transport epithelia these MDR transporters are almost considered ectopical in neurons, this means occurring in an unusual place. RT-PCR analysis of rat brains revealed the neuronal expression of MRP1, MRP3, MRP4 and MRP5 [39], [40]. Pgp also has been localized to a specific neuronal cell type in the hippocampus of mice [41]. More interestingly, both genes, *mdr1a and b*, were transiently overexpressed in hippocampus neurons after a pathological situation with insult [42] pointing to a role of Pgp in neuroprotection.

Immunofluorescence labeling localized MRP1 to the sustentacular cell layer of the rat OE [19] and it would have been of special interest to assess MDR functionality in the whole sustentacular cellular layer. This band is only 6–9 µm small and the sustentacular cells are less contoured than ORNs. As they are intermingled with the ORNs, we have rarely been able to identify them unambiguously but for cyclosporin and MK end-point fluorescence intensities. The sustentacular cells show comparable fluorescence intensities to ORNs, and MK appears to have the largest inhibitory effect. This suggests that both cell types, ORNs and sustentacular cells, contributed to the overall fluorescence in the OE measured in presence of Pgp or MRP inhibitors.

To obtain evidence that the functionally detected transporters are expressed in OE, RT–PCR analysis was performed. Recent studies have detected the presence of mRNA of MRP1, MRP3, MRP4, and MRP5 in adult rat OE [14], [19], [20], [33], but no data are avaible for the OE of mouse or newborn rodents. Our RT-PCR results show that both Pgp encoding genes *mdr1a* and *mdr1b* as well MRP1 are expressed in OE of newborn and adult animals of both species. Despite of differences in the tissue source, this is in agreement with previous results for adult rat OE reported by other authors [14], [19], [20]. The aforementioned investigations included olfactory endoturbinates or used endoturbinates exclusively [20] (personal communication) as olfactory tissue source. As turbinates contain significant amounts of contaminating respiratory cells [43] we exclusively sampled olfactory tissue to perform RT-PCR. Thus the presented results on *mdr1a*, *mdr1b* and MRP1 expression are olfactory tissue-specific for newborn and adult rat and mouse. The perinatal expression pattern of Pgp isoforms and MRP1 does not undergo changes with postnatal maturation as we find all three MDR transporters expressed in adult animals of both species. We do not know, however, if the level of expression is increased during postnatal maturation as our RT-PCR was only qualitative. Physiological modulation during ontogenesis can be hypothesized for these proteins in OE similar to that during mouse and rat brain maturation [44]–[46]. Our results corroborate the results obtained with the functional assay. Both transporters have been fully functional early at birth as the calcein-AM assay has been performed on animals at the day of birth.

If Pgp and MRP transporters are functional in ORNs and in the OE, would their presence contribute to the physiological activity of ORNs, namely to the response to odorants? Comparison of the EOGs and their kinetics provide valuable information about how modifications or experimental manipulations influence the signaling underlying the ORNs response to odorants. We analyzed responses to single odorants and to an odorant mixture exciting a large receptor population. We included two stimuli, KCl and IBMX, which elicit EOGs not mediated by olfactory receptor activation. The elevated KCl response is a measure of general OE excitability, and IBMX, a phosphodiesterase inhibitor leads to an increase in ciliary cAMP thus activating the downstream signal transduction cascade.

Inactivation or reversal of Pgp and MRP function by appropriate inhibitors was associated with a significant reduction in EOG amplitude. Verapamil-mediated Pgp inhibition reduced the amplitude of most odorant responses without significantly affecting the amplitude of IBMX and KCl, indicating the functioning of the cAMP signaling pathway. Based on this observation one may hypothesize that Pgp may be interfering directly with odorants or that interaction takes place at or close to the olfactory receptor site. Other than as for Pgp dependant interaction, the result can be interpreted by virtue of the quality of verapamil as a blocker of voltage-activated calcium channels. The localization of the cellular interaction of the transporters and/or inhibitors with the odorant response can be inferred from the EOG recordings. EOGs record generator potentials which arise mostly in the ciliary parts of the ORNs where the signal transduction process occurs. Contribution may also come from the knob and the upper dendritic part of the ORN. As cilia do not contain voltage-activated channels [47] the lipophilic verapamil has to partition into the knob where L-type calcium channels are localized [48]. They may open in response to odorants and be closed by verapamil thus reducing the EOG amplitude. The Pgp inhibitor cyclosporin only slightly reduced EOG amplitudes indicating no significant interaction with odorant induced events. This is in contrast to its strong inhibitory effect evidenced in the calcein assay. There are two principle differences in the two preparations which could account for this: e.g. the effect may depend on the availability of the inhibitor at its target site. Other than in the calcein assay where cells have been almost directly exposed to the inhibitors, in the EOG experiments they have first to travel across an aqueous layer of mucus to diffuse to their site of action. Different physicochemical properties could influence their passage. A more important difference is that not calcein-AM is the substrate but an unknown Pgp-target molecule. There, inhibitory effects may depend on the combination of transporter-substrate-inhibitor and react differently in diverse assays. Although cyclosporin and verapamil bind with high affinity to Pgp [49], [50] their interaction with Pgp differ [51], [52]. It is also possible that the anatomical localization of Pgp transporters is far from the site of signal transduction, not allowing interaction or electrophysiological EOG recording. Regarding the discrepancies and the lack of specific cyclosporin- and verapamil-mediated effects, it remains unclear if the Pgp transporter is involved in the early part of the olfactory response. To clarify this, further investigations are needed.

Blockade of MRP function by probenecid or MK571 strongly decreased and almost abolished EOG amplitudes of the odorant and IBMX-induced responses. The fact that IMBX-induced responses have also been reduced by MRP inhibitors suggests that the interaction between MRP and/or inhibitors does not occur at the receptor site. One has to consider whether any of the inhibitors may have an effect on EOGs by interacting with one of the elements in the signal transduction chain. Recently. Xie et al, [53] demonstrated that MRP4 inactivation by MK571 and probenecid is associated with reduced cAMP degradation by cellular phosphodiesterases. According to their model, both MK and probenecid acted as phosphodiesterase inhibitors in various MRP4 expressing cell lines. MRP4 and MRP5 are transporters associated with the efflux of cyclic nucleotides like cAMP and cGMP from cells [54], [55]. In ORNs, cAMP has a crucial role in signal transduction and its level is tightly regulated by rapid degradation of phosphodiesterases. Given that MK and probenecid may block phosphodiesterases activity in ORNs this would be similar to a functional ablation of their activity. This effect has been studied in a mouse line with gene disruption for both subtypes of phosphodiesterases identified in ORNs [56]. Contrary to the assumption that the resulting increase in cAMP would lead to an augmentation of the olfactory response, odorant-induced EOGs exhibited significant reduced amplitudes. Also the unspecific phosphodiesterase blocker IBMX reduced odorant–induced current amplitudes in single ORNs when IBMX was applied together with odorants [57]. Such mechanisms of action could explain the reduced EOG amplitudes recorded in presence of MK and probenecid. It is not known if the model of Xie et al. can be extended to other MRPs than to MRP4, and which of the MRPs in ORNs would participate in this process. Our calcein assay does not provide information to which of the MRP isoforms is involved in the fluorescence uptake. Possible contribution may come from MRP1, MRP3, MRP4 or MRP5. While calcein is not a substrate of MRP5, MRP5 activity may be inhibited by MK and probenecid as shown for human MRP5 [58].

There is no simple interpreation to explain such changes in a system where the measured response is the result of complex pathways and feedback mechanisms. Reduced EOG amplitudes are compatible with a faster response termination. The most compatible explanation may be that MRP-like MDR transporters interact with mechanisms in the calcium signaling network. Calcium plays a pivotal role as a third messenger in olfactory transduction and can either potentiate or attenuate the olfactory signal [59]. Calcium can also modulate receptors and reduce olfactory sensitivity [60].

Together, the data presented here have shown that MDR transporters are functional in OE and in ORNs of rodents. They also show how sensory responses to odorants were altered by inactivation/inhibition of MDR function, especially by inhibition of MRP-like transporters. Among proposed functional roles tissue detoxification may be a vital element especially with the olfactory epithelium being the principal site of absorption of inhaled compounds. The interaction of natural and environmental cytotoxic compounds with ABC transporter have been documented in several studies [3], [61]. Inhibition or inactivation of MDR transporters may lead to reduced acuity and in turn modify animal behavior. Besides being detrimental and health threatening to the animal, it may also be beneficial as a continuous exposure may induce up-regulation of the transporters in the olfactory tissue [20]. There is also growing evidence of pheripheral modulation of olfactory signals by input from autonomic innervations by neurotransmitters [62]. MDRs may contribute to uptake and efflux of such substances i.e. adrenaline which modulates signal activities in the olfactory epithelium [63] may be transported by MRP1 as shown for other tissue [64].
